# The effects of extraction of pulpally involved primary teeth on weight, height and BMI in underweight Filipino children. A cluster randomized clinical trial

**DOI:** 10.1186/1471-2458-12-725

**Published:** 2012-08-31

**Authors:** Bella Monse, Denise Duijster, Aubrey Sheiham, Carlos S Grijalva-Eternod, Wim van Palenstein Helderman, Martin H Hobdell

**Affiliations:** 1 Gesellschaft für Internationale Zusammenarbeit (GIZ), Leviste cor Rufino Street, Makati City, Metro Manila, Philippines; 2 Department of Preventive Dentistry, Academic Centre for Dentistry Amsterdam, Gustav Mahlerlaan 3004, Amsterdam, 1081LA, The Netherlands; 3 Department of Epidemiology and Public Health, University College London, Torrington Place 1-19, London, WC1E 6BT, UK; 4 Centre for International Health & Development, UCL Institute of Child Health, 30 Guilford Street, London, WC1N 1EH, UK; 5 Dental Health International Nederland (DHIN), Korte Linschoten OZ 14, Linschoten, 3461 CG, The Netherlands

**Keywords:** Underweight, Weight gain, Growth, Dental caries, Dental decay, Tooth extraction, Dental extraction, Stepped wedge design, Cluster randomized trial, Clinical trial

## Abstract

**Background:**

Severe dental caries and the treatment thereof are reported to affect growth and well-being of young children. The objective of this study was to assess the effects of extraction of severely decayed pulpally involved primary teeth on weight and height in underweight preschool Filipino children.

**Methods:**

Underweight preschool Filipino children with severe dental decay had their pulpally involved primary teeth extracted during a stepped wedge cluster randomized clinical trial. Day care centers were randomly divided into two groups; children from Group A day care centers received treatment as soon as practical, whereas children from Group B day care centers were treated four months after Group A. Clinical oral examinations using WHO criteria and the pufa-index were carried out. Anthropometric measurements were done on both groups immediately before treatment of Group A and at follow-up four months later. Height and weight *z*-scores were calculated using 2006 and 2007 WHO Growth Standards. Multilevel analysis was used to assess the effect of dental extractions on changes in anthropometric measurements after dental treatment.

**Results:**

Data on 164 children (85 in Group A and 79 in Group B), mean age 59.9 months, were analyzed. Both groups gained weight and height during the trial period. Children in Group A significantly increased their BMI (p < 0.001), and their weight-for-age (p < 0.01) and BMI-for-age *z*-scores (p < 0.001) after dental treatment, whereas untreated children in Group B did not. Children in Group A had significantly more weight gain (p < 0.01) compared to untreated children in Group B. However, children in Group A had an inverse change in height gain (p < 0.001). Adjustment for the time interval between the two visits had little effect on the results.

**Conclusions:**

The extraction of severely decayed primary teeth resulted in significant weight gain in underweight Filipino children. Untreated dental decay should be considered an important co-factor affecting child growth and should be considered when planning for interventions to improve child growth.

**Trial registration:**

ISRCTN90779069 http://www.controlled-trials.com/isrctn/isrctn_loa

## Background

In many low- and middle-income countries, the prevalence of untreated dental caries in the primary dentition of young children is high [1,2]. For example, the 2006 Philippine National Oral Health Survey showed that dental caries was universal in six-year-old children [3]. Their mean number of decayed or missing teeth was 8.4, no teeth were filled and 40% of the decay had progressed into an odontogenic infection, such as pulp involvement, abscesses or fistulas.

Poor oral health in children is associated with underweight and failure to thrive. Children requiring multiple extractions of severely decayed teeth had significantly lower body weights than caries-free children [4-6]. Similar results were observed in the Philippines National Oral Health Survey 2006 [7]. Complete dental rehabilitation of underweight children with severe dental decay was associated with an increased rate of weight gain [8-10]. Rate of weight gain may have been related to elimination of dental pain and sepsis that negatively affected children’s ability to eat and sleep. However, no causal relationship between severe dental decay and growth could be deduced because of the design of the studies.

Two randomized controlled trials investigating the impact of dental treatment on body growth have been conducted by [Gemert-Schriks et al. 11] and [Alkarimi 12]. Both studies reported no significant differences in anthropometric outcomes between children receiving or not receiving comprehensive dental treatment.

Based on the conflicting findings of cross-sectional studies and the clinical trials, this clinical trial was planned with the objective of assessing the effects of extraction of severely decayed pulpally involved primary teeth on weight, height, and BMI in underweight preschool Filipino children.

## Methods

### Study population

All children included in the study were attending day care centers in municipalities in the Provinces of Cagayan de Oro and Misamis Oriental, Northern Mindanao, Philippines. The children were aged between 48 and 68 months. They were all underweight and had one or more pulpally infected primary teeth as a result of severe dental decay. Children were considered underweight if their BMI was below the 5^th^ percentile according to CDC Growth Charts. All children were tested for active tuberculosis infection (TB). Children who tested positive for TB were referred to a governmental TB program for treatment and they were not included in the study. Intellectually challenged children were also not included for ethical reasons. None of the children included in the study had systemic medical conditions and/or infectious diseases, according to reports by their parents.

### Ethics statement

All parents or caregivers had signed an informed consent. Written ethical approval for the study was obtained from the Ethics Commission of Xavier University, Cagayan do Oro City.

### Study design

**Figure 1:**
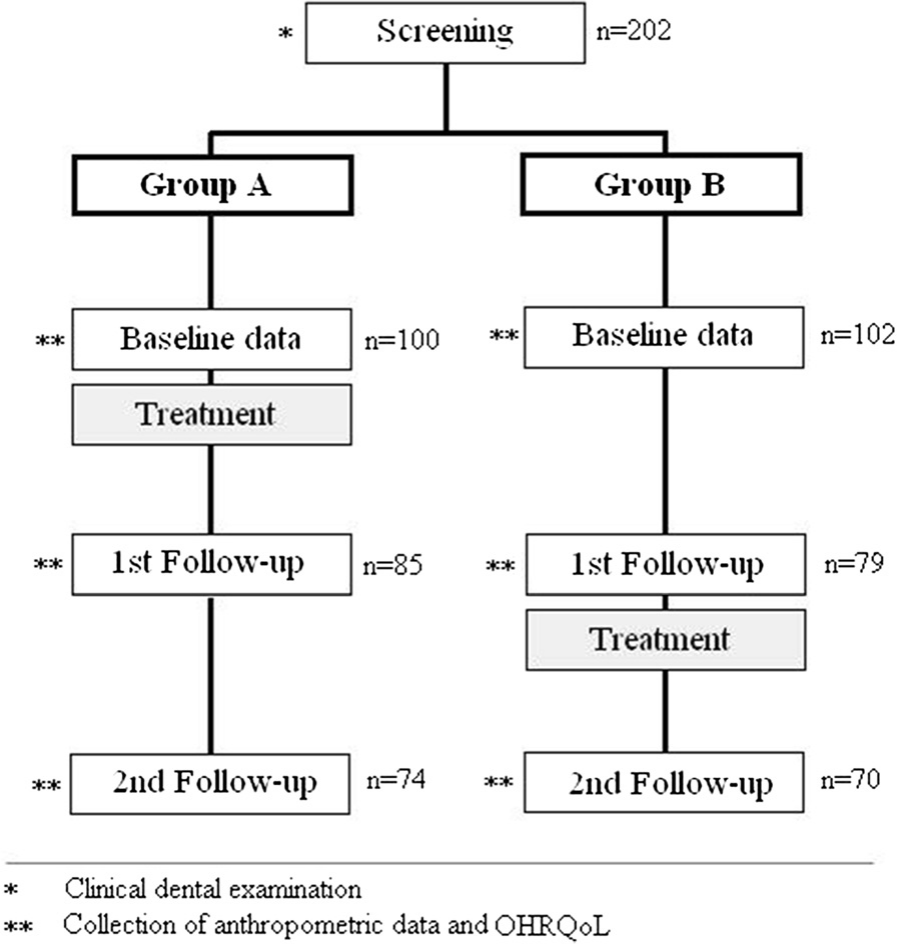
Weight Gain Study Design.

This study is a stepped wedge cluster randomized clinical trial. Thirteen day care centers in ten municipalities in the Provinces of Cagayan de Oro and Misamis Oriental served as the clusters. The day care centers were randomly allocated into two groups; intervention Group A (six clusters) and waiting list control Group B (seven clusters) (Table 1). Children from Group A (*n* = 100) were treated first, and children from Group B (*n* = 102) were treated four months later in the same way as Group A (Figure 1). Treatment involved the extraction of all pulpally involved teeth under local anesthesia and treatment of other carious teeth with silver-diamine-fluoride Arrest of Caries Technique (ACT) [13].

**Table 1:** The randomization of day care centers

**Group A**		**Group B**	
*Day care centers*	*N*	*Day care centers*	*n*
El Salvador (A)	43	El Salvador (B)	17
Laguindingan (A)	21	Laguindingan (B)	11
Opol (A)	4	Opol (B)	32
Cagayan de Oro	16	Libertad	15
Alubijid	13	Initao	14
Talahag	3	Naawan	9
		Manticao	4

### Data collection

Prior to day care center group allocation, all children were orally screened. Socio-demographic data were collected by questionnaire and a face-to-face interview with parents at baseline. Anthropometric measurements, an interviewer-administered Oral Health-Related Quality of Life (OHRQoL) questionnaire, and blood samples were collected for both groups at baseline, four months after treatment of Group A children, and four months after Group B children were treated (Figure 1). OHRQoL data were collected to assess the relation between oral health-related impacts and growth. Blood samples were taken to explore the effect of primary tooth extractions on hemoglobin levels, to test the theory that dental infection affects growth through suppressed erythrocyte production in the bone marrow. This paper reports only on the data relating to primary tooth extractions and its relation with children’s weight, height and Body Mass Index (BMI). Relationships with OHRQoL and hemoglobin levels will be reported in a separate paper.

### Clinical data

Clinical dental data were collected using standard WHO Basic Methods from 1997 [14]. One trained and calibrated general dentist and a recorder carried out all examinations. Children were examined lying in a supine position on carers’ or on examiners’ laps (‘knee to knee’ position) outside the day care centers, using sunlight as the direct light source. As compressed air was not available, cottonwool balls were used to dry the teeth. Caries was scored when ball ended CPI probe could penetrate the dental cavity. Non-cavitated lesions were not recorded.

The dmft/dmfs-index was used to assess dental status. The dmft/dmfs-index expresses caries experience by calculating the number of decayed (d), missing (m) and filled (f) teeth (t) or surfaces (s). In addition, the severity of current dental decay was scored using the pufa-index [15]. The pufa index records the presence of severely decayed teeth with visible pulpal involvement (p), ulceration caused by dislocated tooth fragments (u), fistula (f) and abscess (a). The pufa-score per person is calculated in the same cumulative way as the dmft-score. Children with a minimum of one pulpally involved primary tooth (pufa-score of at least 1) were included in the study.

### Anthropometric data

Weight and height measurements were obtained by a trained nurse in duplicate and the average compounded, following international recommendations [16]. Weight was measured to the nearest 0.1 kg using portable hanging scales (Salter scale, UNICEF procurement), which were calibrated after every five measurements. Standing height was measured to the nearest 0.1 cm using a stadiometer ('Leicester' Model, Children's Growth Foundation, UK).

### Data handling

Weight and height data were transformed to *z*-scores, namely weight-for-age (WAZ), height-for-age (HAZ) and BMI-for-age (BAZ), with the lmsGrowth excel add-in (Medical Research Council, 2008), using the 2006 [17] and 2007 [18] WHO Growth Standards. *Z*-scores allow comparison of an individual’s weight, height or BMI, adjusting for age and sex relative to a reference population, expressed in standard deviations from the reference mean.

Growth, defined as change in anthropometric values between two consecutive time points, was assessed in three ways. First, as the absolute difference between two untransformed anthropometric measurements; second, as the difference between two *z*-scores, therefore controlling for age and sex relative to the reference population; and third, as the difference between two *z*-scores conditional to baseline anthropometric measurements, expressed as conditional growth velocity (CGV). CGV additionally controls for ‘regression towards the mean’, where extreme large or small values at baseline are unlikely to remain extreme at follow-up [19,20]. CGV was expressed as conditional weight, height or BMI velocity (CWV, CHV and CBMIV, respectively), using unexplained residuals [21]. CWV was calculated by regressing WAZ at follow-up against WAZ at baseline, separately by sex. Predicted WAZ was then obtained from the regression. CWV was calculated as the difference between observed and predicted WAZ at follow-up, divided by the standard deviation of those differences. The same procedure was used to calculate CHV and CBMIV. CGV outcomes, namely CWV, CHV and CBMIV, can be interpreted as growth (changes in WAZ, HAZ and BAZ) above or below that is expected given baseline anthropometric measurements, sex and age.

### Statistical analyses

Descriptive analysis of clinical and anthropometric data was carried out using SPSS version 17.0 (SPSS Inc, Chicago, IL, USA). All children with missing data due to loss to follow-up or unrecorded data were excluded from the analysis. Paired *T*-test was used to assess changes in anthropometric measurements between different time points within each group.

In this study, individuals (first level) were nested in day care centers from different municipalities (second level). Therefore, multilevel modeling was used to assess the effect of extraction of severely decayed teeth on growth indicators (weight, height, BMI, WAZ, HAZ, BAZ, CWV, CHV and CBMIV). Multilevel analyses were carried out using R version 2.14.2. (R Foundation for Statistical Computing, Vienna, Austria). For each multilevel model both the fixed effect coefficients and the random effects variances between day care centers are presented, though the interpretation of the results focuses on the fixed effects. The intercept shows the average change in growth indicators of the study population. The regression coefficient reflects the absolute difference in growth indicators in Group A children, relative to children in reference Group B. All models were adjusted for the time interval between baseline and the first follow-up. CGV outcomes already account for the adjustment of age, sex and baseline anthropometric measurements.

For each model, the intraclass correlation coefficient (ICC) was calculated. ICC could be interpreted as the percentage of total variance in growth that is due to differences between day care centers. The remaining proportion is between-individual variation. The level of significance was set at 5%.

## Results

Eighty one percent of the 202 children included in the study (164 children; 85 in Group A and 79 in Group B) were reassessed at the first follow-up four months later; 71.3% (144 children; 74 in Group A and 70 in Group B) completed all stages of the study. Data for 164 children were analyzed (81.2%), after excluding all children with missing data due to drop-out or unrecorded data. The frequent migration of families was the main reason for loss to follow-up.

### Baseline characteristics

The baseline sample of 202 children was aged between 48 and 68 months and had more girls (58%) than boys (42%) (Table 2). Sex was not evenly distributed among the two groups as Group B had significantly more girls. The monthly family income ranged between US$20 and US$320, with an average monthly income of US$99 per family. All children had at least one tooth with severe dental decay. The average pufa-score of the children was 2.3 ± 1.6. They had a mean of 2.0 ± 1.7 teeth with pulp involvement, 0.2 ± 0.5 teeth with a fistula and 0.05 ± 0.02 teeth with an abscess. The average WAZ was −2.1 ± 0.6 *z*-scores (in Group A children) and −2.5 ± 0.8 *z*-scores (in Group B children) below the mean of the reference population. At baseline Group A children were significantly heavier and taller than Group B children.

**Table 2:** Baseline characteristics of children included in the study

**Characteristics**	**Group A (n = 100)**		**Group B (n = 102)**		
	***n***	***(%)***	***n***	***(%)***	p-value*
Male	49	(49)	36	(35)	0.05
Female	51	(51)	66	(65)	
	***Mean* ± *SD***		***Mean* ± *SD***		p-value**
Age (months)	59.9 ± 5.0		59.7 ± 4.7		0.7
Pufa-score	2.3 ± 1.4		2.4 ± 1.8		0.8
Weight (kg)	13.9 ± 1.2		13.2 ± 1.4		< 0.001
Height (cm)	102.2 ± 4.3		100.2 ± 5.2		< 0.01
BMI (kg/m²)	13.3 ± 0.6		13.1 ± 0.7		< 0.01
WAZ (*z*-score)	−2.1 ± 0.6		−2.5 ± 0.8		< 0.001
HAZ (*z*-score)	−1.6 ± 0.8		−1.9 ± 1.0		< 0.01
BAZ (*z*-score)	−1.6 ± 0.5		−1.7 ± 0.7		0.04

* Chi²-test, ** Independent Sample *T*-test.

### Results for both groups at first follow-up

Group A children had an average of 2.4 ± 1.4 teeth extracted, ranging from one and nine extractions per child. The mean time interval between baseline and the first follow-up was significantly greater for Group A than Group B children (4.0 ± 0.7 and 3.5 ± 0.6 months for Group A and B respectively, p < 0.001).

All children significantly gained weight and height between baseline and the first follow-up (Table 3). However, children in Group A also had significant increases in BMI, WAZ and BAZ, whereas dentally untreated children in Group B did not. On the other hand, Group B children showed a significant increase in HAZ at the first follow-up, whereas the average HAZ in Group A children declined by 0.1 *z*-scores.

**Table 3:** Anthropometric measurements at baseline, at first follow-up and for Group B at second follow-up

	**Group A**		**Group B**		
	**Baseline**	**1**^**st**^**follow-up**^**a**^**Treated**	**Baseline**	**1**^**st**^**follow-up**^**b**^**st**	**2**^**nd**^**follow-up**^**c§**^**Treated**
	***mean (95% C.I.)***	***mean (95% C.I.)***	***mean (95% C.I.)***	***mean (95% C.I.)***	***mean (95% C.I.)***
Weight (kg)	13.9 (13.6, 14.2)	14.9 (14.6, 15.2)**	13.3 (13.0, 13.6)	13.8 (13.5, 14.1)**	15.3 (14.8, 15.8)**
Height (cm)	102.4 (101.5, 103.3)	103.9 (102.9, 104.9)**	100.4 (99.3, 101.5)	102.4 (101.3, 103.5)**	104.7 (103.3, 106.0)**
BMI (kg/m²)	13.3 (13.2, 13.4)	13.8 (13.6, 14.0)**	13.1 (13.0, 13.2)	13.2 (13.1, 13.3)	13.9 (13.6, 14.2)**
WAZ (*z*-score)	−2.1 (−2.2, -2.0)	−1.8 (−1.9, -1.7)*	−2.4 (−2.5, -2.3)	−2.3 (−2.5, -2.1)	−1.9 (−2.1, -1.7)**
HAZ (*z*-score)	−1.5 (−1.7, -1.3)	−1.6 (−1.8, -1.4)**	−1.9 (−2.1, -1.7)	−1.8 (−2.0, -1.6)*	−1.8 (−2.1, -1.5)
BAZ (*z*-score)	−1.6 (−1.7, -1.5)	−1.2 (−1.3, -1.1)**	−1.7 (−1.8, -1.6)	−1.7 (−1.8, -1.6)	−1.1 (−1.3, -0.9)**

Paired *T*-test, * < 0.01 ** < 0.001.

^a^*n* = 85, ^b^*n* = 79, ^c^*n* = 63, ^§^ 7 missing.

Multilevel analyses show that children in Group A had significantly greater weight gain after dental treatment, compared to untreated children in Group B (Table 4). This difference was observed whether weight gain was assessed using untransformed data, *z*-scores or as CGV. For example, on average children in Group B gained 0.49 kg between baseline and the first follow-up. However, children in Group A had an additional average weight gain of 0.46 kg compared to Group B children. Similar results were found for BMI values. Conversely, untreated children in Group B had more height gain compared to children in Group A, although this difference was not significant for height and CHV.

**Table 4:** Anthropometric changes, comparing Group A with Group B*, between baseline and 1st follow-up

	**Fixed effects**					**Random effects**		
	***Intercept***	***95% C.I.***	***β***^**†**^	***95% C.I.***	***p-value***	***1***^***st***^***level variance***	***2***^***nd***^***level variance***	***ICC (%)***
Δ Weight (kg)	0.49	(0.31, 0.67)	0.46	(0.20, 0.72)	< 0.01	0.42	0.01	1.7
Δ WAZ (*z*-score)	0.02	(−0.06, 0.10)	0.23	(0.11, 0.35)	< 0.01	0.14	0.00	0.0
CWV (SR)	−0.39	(−0.64, -0.14)	0.70	(0.33, 1.07)	< 0.01	0.90	0.02	2.5
Δ Height (cm)	1.98	(1.57, 2.42)	−0.61	(−1.28, 0.06)	0.06	2.00	0.14	9.0
Δ HAZ (*z*-score)	0.05	(−0.04, 0.14)	−0.14	(−0.28, 0.00)	0.04	0.09	0.01	2.0
CHV (SR)	0.13	(−0.18, 0.44)	−0.35	(−0.81, 0.11)	0.12	0.91	0.08	7.3
Δ BMI (kg/m²)	−0.05	(−0.20, 0.10)	0.56	(0.34, 0.78)	< 0.001	0.44	0.00	0.0
Δ BAZ (*z*-score)	−0.02	(−0.12, 0.16)	0.49	(0.29, 0.69)	< 0.001	0.35	0.00	0.0
CBMIV(SR)	−0.46	(−0.67, -0.25)	0.88	(0.57, 1.19)	< 0.001	0.85	0.00	0.0

* Multilevel model, adjusted for the time interval between baseline and 1^st^ follow-up.

^†^ Regression coefficient: the reference is Group B.

SR: Standardized residuals.

The random effects variances show that 2.0% to 9.0% of the variance in height gain occurred at the day care center level and that 91.0% to 98.0% of the variance in height gain occurred at the individual level. Zero to 2.5% of the variation in weight gain and none of the variation in BMI occurred between day care centers. Furthermore, the effect of dental treatment on growth indicators was adjusted for the time interval between baseline and the follow-up measurement. The adjustment did not result in major changes of the results.

### Results for Group B at second follow-up

Group B children had an average of 2.0 ± 0.9 teeth extracted and were re-examined 5.1 ± 0.5 months after dental treatment. Children in Group B showed significant changes in weight, height, BMI, WAZ and BAZ after dental treatment (Table 3). They also showed a decline in HAZ after receiving dental treatment although it did not reach statistical significance. The growth pattern observed was similar to that recorded for Group A children after they had been treated (Table 3).

## Discussion

The results of this cluster randomized clinical trial show a clear effect of extraction of severely decayed primary teeth on weight gain in underweight Filipino children, although there was no increase in height gain. The findings were consistent with previous non-controlled studies [8,10]. However, our results differ from those of [Gemert-Schriks et al. 11] and [Alkarimi 12] who reported insignificant changes in mean anthropometric outcomes of dentally treated children compared to untreated controls, although the changes they reported in weight showed the same trend as those reported here. The difference in findings could be attributed to the fact that not all the children in their studies were underweight and children had less severe dental decay than in the present study. That could result in treatment having effect on weight gain, as there was less dental sepsis and dental impacts to eliminate. Another factor that affected the outcomes of Gemert-Schriks’ study is that dental infection was not eradicated; children developed new severe caries lesions during the course of their study.

There are several plausible mechanisms for the effect of dental extractions of teeth with severe caries on increased velocity of weight gain [22]. Untreated severe dental decay and the resulting pain may contribute to disturbed sleeping habits and inadequate caloric intake of children. Inadequate sleep may also affect secretion of growth hormones [23] or may cause excessive energy expenditure, while impacts on eating may affect quality and quantity of nutritious food consumed. These theories are partially supported by findings from [Anderson et al. 24] and [Acs et al. 25] that showed that after dental treatment significant improvement was noted in the children’s pain and discomfort experience, sleep patterns and in their appetite and quantity of foods eaten. Another explanation is that dental inflammation from pulpitis and dental abscesses suppresses growth through a metabolic pathway by reducing hemoglobin as a result of depressed erythrocyte production in the bone marrow [26]. However, based on currently available research, no theory can be confirmed or excluded.

In the present study some children’s WAZ has deteriorated. This suggests that other medical, social, or environmental factors may have interacted in the association between severe dental decay and growth. Most of the children participating in this study were from very deprived municipalities, where access to food has the highest priority for a large segment of the population and could contribute to parental stress [27]. Several studies emphasized the importance of parental stress on the child’s failure to thrive [28]. Other factors, such as poor environments, parasitic infections and dietary factors are more likely to have a stronger influence on weight gain than dental treatment. For example, the prevalence of soil-transmitted helminths infestation in under-5-year-olds in the Philippines ranged from 49% to 93% [29]. Worm infestation has harmful impacts on nutrition as parasites retard growth through decreased nutrient intake and disturbed metabolism. Other medical factors such as anemia, infectious diseases, respiratory tract infections and diarrhea can play an important role in weight gain [28]. These factors were not assessed in this study, and may result in an underestimation of the association between severe dental decay and growth. However, it is unlikely that those factors alone may explain the observed differences between Group A and B, since these factors were evenly distributed between the two groups.

Another factor that should be noted is that a small number of children in the study had a higher pufa-score than the number of teeth extracted during the intervention, because they required more extractions than was considered acceptable for such young underweight children. The remaining teeth with pulp involvement that were not extracted may explain why there was deterioration in the weight-for-age of some children even after (partial) dental treatment.

A notable finding in this study was that both Group A and Group B children significantly decreased in HAZ after they were dentally treated, while Group B children significantly improved in HAZ in the months before they received treatment. Height, however, takes more time to change than weight. Given the short time span of four months between dental treatment and follow-up, the significant HAZ changes could potentially be explained as saltation and stasis, whereby infant growth follows a series of rapid growth spurs (saltation), separated by periods of stasis [30]. This indicates that children first accrue the necessary mass by putting on weight to subsequently grow in height. This may be an explanation for some of the significant fluctuations in HAZ observed in this study.

One of the main strengths of this study was that the data were derived from a cluster randomized clinical trial, with data collected before and after the intervention. The findings show the benefit of the dental treatment on weight gain. A novel approach of this study is that growth indicators were analyzed as raw values, *z*-scores (standardized for age and sex from a reference population) and as conditional growth velocity (controlling for initial anthropometric measurements). Some potential limitations of this study should be taken into account. They include the relatively small sample size and the considerable number of children lost to follow-up. However, the 81.2% follow-up rate is satisfactory, especially considering the difficult conditions prevailing at study sites.

Further research is needed to investigate the effects of severe dental decay in children on body constitution and growth and the causal mechanisms for their relationship. Future studies need to investigate the metabolic pathways and incorporate parameters related to general health, infectious diseases, psychosocial relationships and environmental factors. Ideally, the measurements of these variables should precede the measurements of the outcomes, namely, ‘weight and height gain’, to assess temporality. The time span between dental treatment and the assessment of anthropometric measures should be prolonged and anthropometric indicators should be regularly monitored in order to investigate the effect of dental treatment on height.

## Conclusions

The findings of this study show that the treatment of severe dental caries significantly improves growth of underweight young children. This important relation between severe dental caries and child body constitution and general health must be investigated further since the burden of untreated dental caries is particularly high in deprived children in low- and middle-income countries. Vertical programs to improve nutritional status of underweight children will fail if they do not address the underlying reasons, and untreated dental decay is one of them. Feeding programs around the globe have incorporated deworming strategies as a prerequisite prior to feeding. If the demonstrated significant impact of oral health on body constitution is better understood, emphasis on prevention of dental decay and basic oral care need be one of the priorities of integrated health promotion programs as well as become part of feeding strategies to enhance the well-being of the millions of underweight children worldwide.

## Abbreviations

ACT: Arrest of Caries Technique; BAZ: BMI-for-age; BMI: Body Mass Index; CBMIV: Conditional BMI Velocity; CGV: Conditional Growth Velocity; CHV: Conditional Height Velocity; CPI: Community Periodontal Index; CWV: Conditional Weight Velocity; dmft: Decayed Missing and Filled Teeth; dmfs: Decayed Missing and Filled Surfaces; HAZ: Height-for-age; ICC: Intraclass Correlation Coefficient; OHRQoL: Oral Health Related Quality of Life; pufa: Presence of severely decayed teeth with visible pulpal involvement Ulceration caused by dislocated tooth fragments Fistula Abscess; TB: Tuberculosis; WAZ: Weight-for-age; WHO: World Health Organization.

## Competing interests

The authors declare that they have no competing interests.

## Authors' contributions

Conception and study protocol (BM, AS, MHH), study implementation and data collection (BM, MHH), data analysis (DD, CSGE), interpretation of findings (BM, DD, AS, CSGE, MHH), drafting of the initial manuscript (DD), revision of the manuscript (BM, DD, AS, CSGE, WvPH, MHH), agreed to the final version of the manuscript (BM, DD, AS, CSGE, WvPH, MHH).

## Pre-publication history

The pre-publication history for this paper can be accessed here:

http://www.biomedcentral.com/1471-2458/12/725/prepub
